# Long-Term Quality of Life in Adult Patients with Strabismus after Corrective Surgery Compared to the General Population

**DOI:** 10.1371/journal.pone.0166418

**Published:** 2016-11-15

**Authors:** Meiping Xu, Huanyun Yu, Yuanyuan Chen, Jinling Xu, Jingwei Zheng, Xinping Yu

**Affiliations:** The Eye Hospital of Wenzhou Medical University, Wenzhou, Zhejiang, China; Soochow University Medical College, CHINA

## Abstract

**Purpose:**

To evaluate the status of and factors associated with long-term health-related quality of life (HRQOL) in adult patients with strabismus following corrective surgery.

**Methods:**

Prospective cross-sectional study. A total of 122 adults who underwent corrective surgery and were followed up for at least 1 year were recruited. Pre- and post-operative HRQOL were evaluated using the Chinese version of the Adult Strabismus 20 (AS-20). Demographics and clinical characteristics were recorded. Eighty-nine age-matched, normal individuals without a history of strabismus were recruited as a control group.

**Results:**

AS-20 scores improved significantly in the psychosocial subscale and total scale after surgery for all122 patients, but not in the function subscale. HRQOL was better in the successful cases than in the non-successful cases (p<0.005). Subjects who recovered stereo function had better HRQOL than those who did not (p<0.01). Compared to the control population, the patients had poorer HRQOL post-operatively, with only approximately 30% of the subjects having scores within the normal threshold scores. The self-sense of a lack of ocular deviation and a successful surgical outcome were significant factors associated with post-operative HRQOL status.

**Conclusions:**

HRQOL, as evaluated by AS-20 scores, improved in the patients after surgery but was worse than that in the general population. Successful surgical outcomes and a sense of good alignment were the main factors that correlated with increased post-operative HRQOL. Positive assessments of surgical results by patients may benefit post-operative HRQOL.

## Introduction

Strabismus affects health-related quality of life (HRQOL) in adults and children in varied ways, including psychosocially and functionally (self-sense, confidence, and life-style preferences) [1–9]. In patients with strabismus, the factors affecting patient HRQOL are related to strabismus characteristics[9–11]; demographics[12]; psychosocial parameters, such as mood, depression, and self-esteem; and understanding of strabismus[13–15]. HRQOL can be improved after corrective surgery in both the short term (6 weeks to 90 days) and long term (1 year to 18 months),as assessed via the Adult Strabismus-20 (AS-20), the Amblyopia and Strabismus Questionnaire (A&SQ), and other generic questionnaires[16–21]. Strabismus surgery in adults has been suggested as a very cost-effective treatment to gain quality-adjusted life years[22,23].Improvement in HRQOL following corrective surgery is associated with various factors, including female gender[13],better alignment after surgery[16,17,24], larger deviation changes[17], and less social anxiety[21].However, it should be noted that most of the above referenced studies evaluated subjects shortly after surgery (7 weeks to 3 months)[17,21,24], without a strong focus on clinical characteristics and demographics and with no comparison to a normal general population.

Psychosocial variables, such as general anxiety, social anxiety, social avoidance and depression, were found to remain worse in adult patients than the corresponding normative values after corrective surgery, although they did improve significantly after surgery[5].The long-term status of HRQOL in adult patients following surgery in relation to that of the general population remains unknown. Furthermore, additional studies are needed to identify the factors affecting long-term post-operative HRQOL, which would allow physicians to better understand patients’ thoughts regarding surgery, thereby leading to better post-operative HRQOL.

The AS-20 was specifically developed to evaluate the impact of strabismus on adults[25].The normal threshold score on the AS-20 is 84 for the general population[26]. The reliability of the AS-20 is good based on test–retest evaluation, and the questionnaire has been recommended for use when assessing changes related to HRQOL in patients with strabismus due to its greater sensitivity than the National Eye Institute Visual Function Questionnaire (NEI-VFQ-25)[26].The AS-20 is also believed to be uninfluenced by other bodily factors when used for patients with strabismus[14].The Chinese version of theAS-20 (CAS-20) has been shown to possess satisfactory reliability and validity and is believed to be a useful tool for studying HRQOL in Chinese patients and for providing comparisons with individuals not affected by strabismus[27].

In the present study, we evaluated the HRQOL of adults with strabismus who underwent corrective surgery and had more than one year of follow-up using the Chinese version of the AS-20. We compared these findings with an age-matched, general population. Factors correlating with post-operative HRQOL were also evaluated.

## Materials and Methods

### Patients and Methods

This was a prospective study of 122 adult patients with strabismus who underwent corrective surgery at the Eye Hospital of Wenzhou Medical University between January 1, 2011 and December 31, 2011. All patients underwent post-operative follow-up for at least 1 year (mean, 18 months; range, 12 to 24 months).Before surgery, 86 patients had exotropia, 34 had esotropia, and 31 patients exhibited vertical deviation. Patients were excluded from the study if they could not read or understand Chinese or if they had associated facial deformities, neurologic disorders, or any significant medical problems. The control group was recruited over the same study period and included 89 age-matched adults (mean, 28.9 years; range,17-58years) with no history of strabismus, with a best corrected visual acuity (BCVA) better than 20/30 in either eye, with no more than 10 prism diopters (PD) of horizontal phoria and 5 PD of vertical phoria by the prism and alternate cover test (PACT), and with no self-sense of ocular deviation. The study was approved by the Eye Hospital of Wenzhou Medical University Ethics Committee and adhered to the tenets of the Declaration of Helsinki. All participants provided written informed consent to participate in this study, and the ethics committee approved this consent procedure.

#### Clinical assessment and questionnaire administration

Full pre- and post-operative orthoptic measurements were recorded. Patient age, gender, education level, occupation, diagnosis, size of deviation, extraocular movements (EOMs), and binocular function at survey time were recorded. Eye position was determined using the cover test, and ocular deviation was measured using the PACT at distance of 5 m and at near of 40 cm. Combined (|dev|) was calculated using the horizontal (dev_h_) and vertical (dev_v_) deviations as |dev| = √ (dev_h_^2^ + dev_v_^2^)[17]. Sensory fusion was tested using the Worth 4 dot test, and stereo acuity was tested using TNO stereopsis tests.

The CAS-20 consists of 20 items in 2 subscales: 10 in a psychosocial subscale and 10 in a function subscale. For each question, a5-point Likert-type scale is used for responses as follows: “never”(score 100), “rarely” (score 75), “sometimes” (score 50),“often” (score 25), and “always” (score 0). A psychosocial score (10 items) and a function score (10 items) are calculated as the mean of all answered items and can range from 0 to100 (worst to best HRQOL)[27]. The patients completed the CAS-20 questionnaire at their pre-operative and last-visit post-operative assessments. The questionnaire was completed before any clinical examination was performed at each visit. The patients were instructed to respond while wearing their habitual refractive correction, including a prism if prescribed.

#### Classification of post-operative measurements

Post-operative success required no diplopia/visual confusion (or only “rare”) for straight ahead distance or for reading, with no more than10 PD of horizontal and 5 PD of vertical tropia in the primary position at distance and near[28].

Post-operative eye position was defined as orthophoria/phoria, intermittent tropia or constant tropia during the cover test.Table 1 shows the classification of associated factors assessed at the last visit.

**Table 1:** Classification of associated factors.

Factor	Classification
EOM(extraocular movement)	Safe, mild overaction or underaction and obvious overaction or underaction
Sensory fusion	Normal or abnormal (suppression or diplopia)
Stereo acuity	Normal stereo (≦60 sec arc), partial normal stereo (120–480 sec arc) or none (> 480 sec arc)
Visual deficit	Defined as one or both eyes with a BCVA less than 20/60
Sense of deviation	Reviewed as “no deviation”, “still have some deviation”, and “still have obvious deviation”
Education level	Higher (college education and upper), secondary (community college and high school), primary (middle school and primary school) and illiterate
Occupation	Work with people (e.g., teacher or salesperson) and work alone (e.g., technician or cook)

Because the HRQOL data did not follow a Gaussian distribution in the 89 control subjects, we defined the normal threshold score for theCAS-20 in the controls as the 5th percentile, as described by Hatt et al (Table 2)[26].

**Table 2:** Percent scoring within the normal threshold before and after surgery.

	Psychosocial subscale	Functional subscale	Total scale
Normal threshold based on 5th percentile	87.5	82.5	86.0
Pre-operative	4%	15%	5%
Post-operative	31%	22%	27%
Successful group	38%	24%	32%
Non-successful group	12.5%	15.6%	12.5%
Comparison between pre-and post-operative	X^2^ = 30.8,*p*< 0.01	X^2^ = 1.98,*p* = 0.2	X^2^ = 21.97,*p* < 0.01

### Statistical analysis

Pre- and postoperative CAS-20 scores were compared using the Wilcoxon signed-rank test. This test was used to compare differences between CAS-20 scores in the patients and control subjects, between the successful and unsuccessful groups, between the group with stereo function and that without stereo function, between the group with sensory fusion and that without sensory fusion, and between the group with a “sense of deviation” and that without a “sense of deviation”. An independent t test was used to compare improvements in CAS-20 scores and subscales between the successful and non-successful groups. A paired t test was used to compare |dev| before and after surgery. The relationship between postoperative outcomes and demographics withCAS-20 scores was assessed via multivariate analysis of variance. All statistical analyses were performed using the SPSS 19.0 software package (SPSS Inc., Chicago, IL, USA).P-values<0.05 were considered statistically significant.

## Results

### Basic data and measurements

The mean age of the patients at the last visit was 27.7 years (range, 17–65 years), and 56 (46%) were female. The median visual acuity was 20/20 (range,20/100 to 20/20) in the better eye and 20/30 (range, light perception to 20/20) in the worse eye. Forty-four cases had visual deficits in one or both eyes.Table 3 shows the pre-operative data for the122 patients: 86 patients had exotropia, 34 had esotropia, and 31 patients exhibited vertical deviation. Among the patients, 91 underwent uniplanar surgery (86 horizontal, 4 vertical and 1 torsional) and31 underwent multiplanar surgery, both horizontal and vertical.

**Table 3:** Basic data for thepatients and controls.

		Pre-operative	Post-operative	Control
Eye position	Orthophoria/phoria	0	86 (70%)	89
	Intermittent exotropia	29 (24%)	14 (12%)	0
	Constant tropia	93 (76%)	22 (18%)	0
Deviation (prism diopters, PD)	Horizontal	48.6 ± 23.5	6.3± 9.2	0.5 ± 1.3
	Vertical	3.0 ± 6.0	1.0 ± 2.6	0
	Combined	49.8 ± 22.2	6.9 ± 9.2	0.5 ± 1.3
Diplopia	With	7 (6%)	11 (10%)	0
	Without	115(94%)	111 (90%)	89
EOM(extraocular movement)	Normal	52 (43%)	85 (69%)	89
	Abnormal	70 (57%)	38 (31%)	0
Worth 4 dot	Normal	2 (1%)	43 (35%)	81 (91%)
	Abnormal	120 (99%)	79 (65%)	8 (9%)
Stereo	Normal	0	19 (16%)	55 (62%)
	Partial	3 (2%)	21 (17%)	28 (31%)
	No	119 (98%)	82 (67%)	6 (7%)

Post-operative outcomes are shown in Table 3. The post-operative |dev| was significantly less than the pre-operative |dev| (t = 22.5, P<0.001). Post-operatively, 76(63%) patients had a self-sense of no deviation, while 46 patients had a self-sense of some deviation or obvious deviation. For the 90 patients who experienced a successful outcome, 68 reported no sense of deviation and 22 reported a sense of deviation.

### HRQOL status followingcorrective surgery

**Fig 1:**
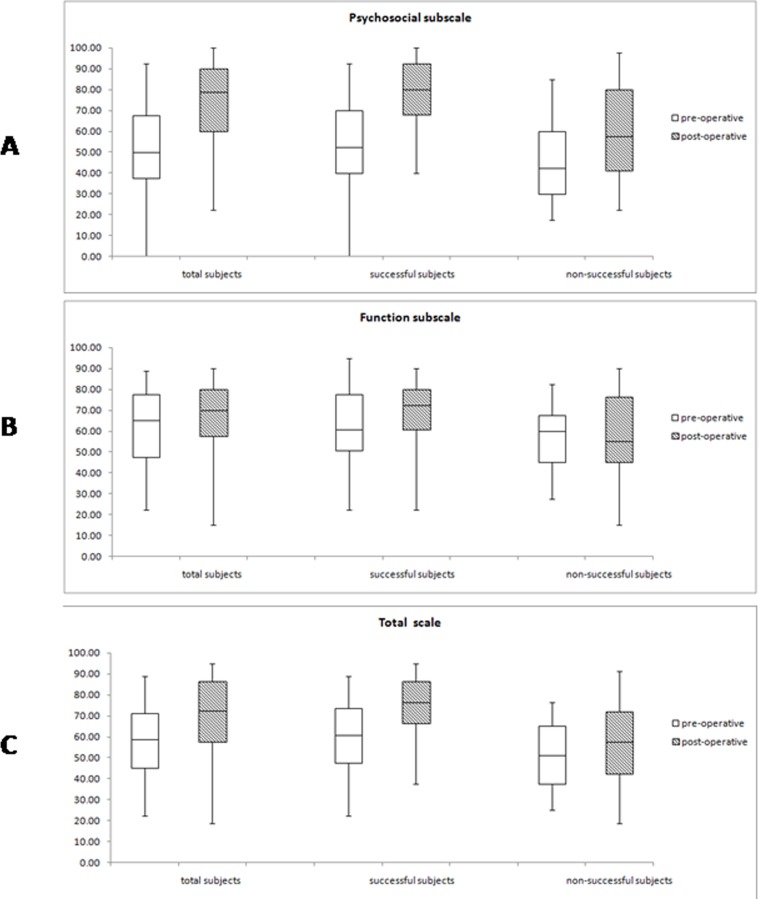
Pre-and post-operative AS-20 scores. A: Psychosocial subscale, B: Function subscale, C: Total scale.

CAS-20 scores improved significantly after surgery in the psychosocial subscale and total scale, but not in the function subscale (Fig 1). The 90 patients with a successful outcome had better scores in both subscales and the total scale than the 32 patients with unsuccessful outcomes (Fig 1, p<0.05).

**Fig 2:**
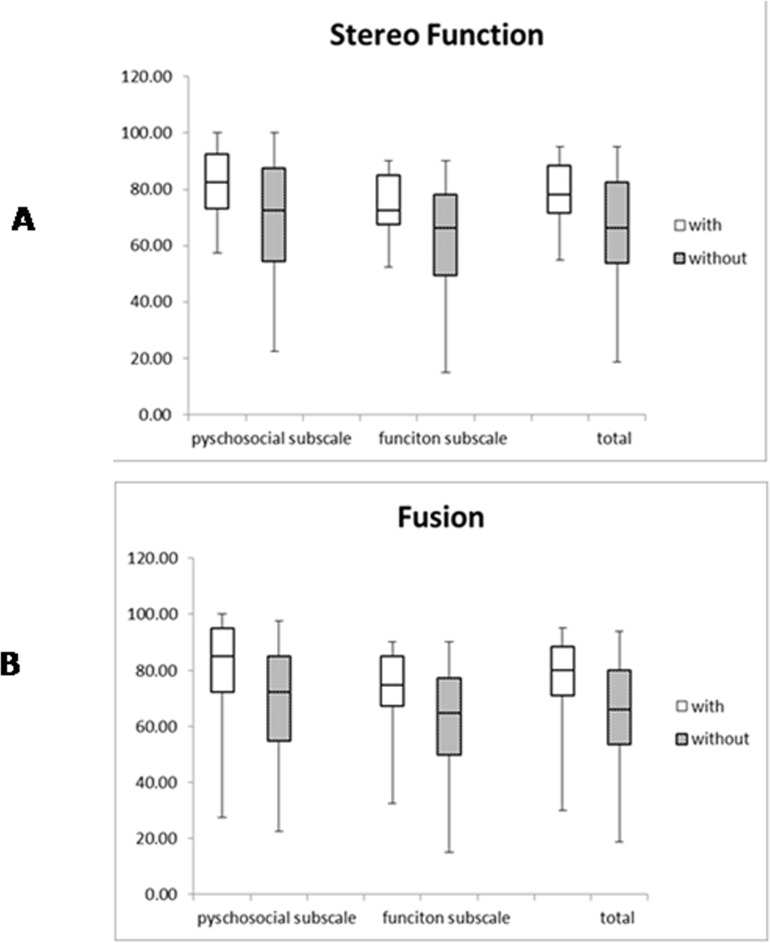
Comparison of binocular function scores between subjects. A: With stereo function and without stereo function, B: With fusion and without fusion.

**Fig 3:**
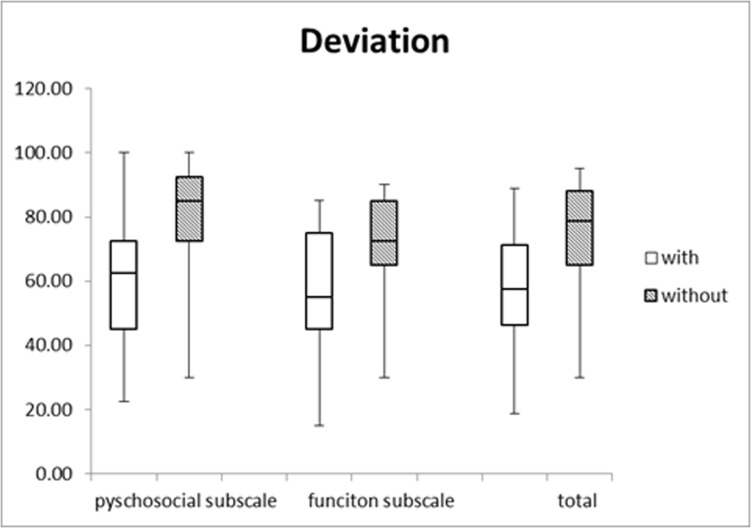
Comparison of scores between subjects with a self-sense of ocular deviation and without a self-sense of ocular deviation.

Better post-operative scores in both subscales and the total scale were observed in the 40 patients with normal or partial stereo function than in the 82 patients without stereo function (Fig 2, p<0.01). Additionally, better scores in both subscales and the total scale were observed in the 43 patients with sensory fusion than in the 79 patients without sensory fusion (Fig 2, p<0.001) and in the 76 patients with no “sense of deviation” than in the 46 patients with a “sense of deviation” (Fig 3,p<0.001). Furthermore, among the 90 patients with successful outcomes, the 68 patients without a sense of deviation had higher scores than the 22 patients with a sense of deviation (Z = 3.01, p = 0.003).

### Comparison of HRQOL between patients with strabismus and controls

The median score on the psychosocial subscale was 78.8 (95% CI: 35–100), the median score on the function subscale was 70 (95% CI: 32.9–90.0), and the median total score was 72.5(95% CI: 37.7–94.8) in the patients after surgery. These values were less than those of the control group, which scored medians of 100 for the psychosocial (95% CI:97.3–99.0), function (95% CI:95.8–98.7) and total scales(95% CI:96.6–98.8) (p<0.001). The total scores were also significantly lower for the 90 successful patients than for the controls (p<0.001). Table 2 shows that 27% of the patients had post-operative HRQOL scores that were within the normal threshold.

### Factors associated with post-operative HRQOL

Table 4 shows the factors associated with post-operative HRQOL, as determined by multivariate analysis of variance. Post-operative orthophoria/phoria, surgical success and a self-sense of not having deviation were significantly associated with better psychosocial subscale scores. Younger age was related to better function subscale scores. Self-sense of no deviation and surgical success were the factors that correlated significantly with a better total scale score.

**Table 4:** Factors associated with HRQOL.

Source	Psychosocial subscale		Function subscale		Total scale	
	F	p	F	p	F	p
Intercept	29.813	.009	93.603	.000	42.349	.001
Age	.027	.871	4.156	.045	.924	.339
Self-sense of deviation	6.354	.014	2.556	.114	16.032	< .001
Gender	.140	.710	1.723	.194	.006	.937
Education level	1.835	.149	1.207	.314	1.256	.294
Occupation	.508	.478	2.410	.125	.184	.669
Pre-operative diplopia	.166	.685	.454	.502	.083	.773
Post-operative position	5.937	.017	.220	.641	3.248	.075
Successful outcome	5.592	.006	.368	.693	3.571	.032
Visual deficit	.318	.575	.286	.595	.004	.951
EOM(extraocular movement)	.874	.422	2.418	.097	2.769	.068
Post-operative diplopia	.658	.420	1.279	.262	.064	.801
Sensory fusion	.150	.700	.000	.993	1.470	.228
Stereo acuity	.205	.815	.879	.420	.666	.516

## Discussion

Similar to previous studies[16–19],the adults with strabismus who were included in the present study showed an improved long-term HRQOL after surgery. However, the HRQOL of these patients, even those that obtained ocular alignment and normal binocular function, remained worse than that of the control population, with only approximately 30% of subjects presenting scores within the normal threshold score. A self-sense of not having ocular deviation and a successful surgical outcome (ocular alignment and without diplopia) were the main factors associated with better HRQOL after surgery.

The pre- and post-operative AS-20 scores for our subjects were comparable to those of previous studies[16,17,26].Additionally, the percentages of scores below normal in the psychosocial subscale and total scale before surgery were similar to those of previous reports[26].The percentage of scores below normal in the functional subscale was adjusted to 55% in our subjects after we applied a score of 70 as the normal threshold based on a previous study; this adjusted percentage was similar to that reported in the study[26].

Long-term negative effects, such as poor self-image and strained interpersonal relationships, have been reported for patients growing up with noticeable strabismus, and these effects often persist through adolescence and adulthood[2,29]. Therefore, although psychosocial variables generally improve after surgery[2,4,30], patients may still exhibit worse HRQOL than the general population with respect to these variables. This may explain why the patients who experienced successful outcomes in the present study still had worse HRQOL than the general population after surgery. It would be worthwhile to evaluate whether HRQOL is better for adults who undergo corrective surgery at a young age versus during adulthood.

As expected, a successful surgical result was the main factor associated with post-operative HRQOL. This is supported by previous studies that found that patients with successful alignment obtained better HRQOL than non-successful subjects[3–5,16–19]. However, Koc et al reported that patients with restored binocular function post-operatively did not show improved HRQOL scores compared to those without binocular function[31]. In our study, patients who recovered binocular function after surgery had better scores than those without binocular function in both subscales. The above finding is also supported by a previous report that patients without binocular inhibition had better HRQOL than those with inhibition[11].

A self-sense of whether deviation exists was one of the most important factors associated with post-operative HRQOL and psychosocial scale scores in our study. There are always some discrepancies between patients and physicians in terms of outcome assessment[32].Satterfield et al found that, although most patients’ lives improved following surgery, more than 80% of patients felt that their eyes were still not perfectly straight[2]. It has been suggested that patients’ social anxiety levels correlate with their post-operative HRQOL[21]. Furthermore, psychosocial characteristics were suggested to play a more important role in well-being than clinical characteristics, as patients who viewed strabismus as a major determining factor in their lives experienced poorer HRQOL[15]. In our study, approximately 24% of the successful subjects still reported a sense of deviation, which may impact their HRQOL. This implies that we should comprehensively consider patient self-esteem and psychosocial characteristics during treatment to obtain better satisfaction and quality of life. As Granet suggested, it is reasonable to conclude that some patients find that obtaining a normal appearance is more important due to social responses to strabismus[33], and physical characteristics should be given the same value as functional characteristics in strabismus treatment[34].

Psychosocial subscale score improvement was greater in the successful group than the non-successful group, but no such improvement was noted for the functional subscale. This contrasted with previous studies reporting that both subscales improved more in successful patients after surgery[16,17,35]. This discrepancy maybe because almost all of the patients in our study had no diplopia before surgery, which would result in poorer psychosocial subscale scores on the AS-20[26].It has also been suggested that the sensitivity of the questionnaire should be modified to include tasks requiring fine stereopsis[31].

Previous studies have shown that female gender, lower socioeconomic status and larger deviation changes are associated with HRQOL pre-operatively and/or post-operatively[12,17,36].We found no significant correlation between HRQOL and age, education level, occupation type, gender, marital status, or direction of deviation. However, socioeconomic status and social anxiety were not reviewed in the current study.

There were some limitations to this study. First, the AS-20 is a specialized questionnaire designed for adults with strabismus, which may have affected the evaluation of HRQOL in the general population compared to the patient population, as many of the control subjects had a maximum score of 100 due to the ceiling effect[25,27]. This ceiling effect may result in lower sensitivity when comparing HRQOL in subjects with a history of strabismus to the general population, as we found in the current study, despite the fact that we compared HRQOL against a normal threshold. It may be better to combine the AS-20 with a generic questionnaire, such as the NEI-VFQ-25.Successful patients may have similar scores on the NEI-VFQ-25 to those of the general population; indeed, a previous study reported that an entire cohort of non-diplopia patients had normal NEI-VFQ-25 scores before surgery and thus had little room for improvement after surgery[24,26,37].Second, as HRQOL evaluates an individual’s overall well-being and life experience, the assessment varied widely between individuals due to many factors, including physical, psychological, personality, social and environmental factors[10,13,35].Adults with childhood intermittent exotropia have been reported to exhibit a higher prevalence of mental illness than controls[20].Social anxiety levels have also been related to post-operative HRQOL in adult patients[21]. Based on the above, further evaluations combining evaluations of strabismus clinical features with assessments of personality changes and overall mental health are warranted.

## Conclusions

After undergoing corrective surgery, adult patients with strabismus presented with worse HRQOL, as evaluated by the AS-20, than a general population. A successful surgical outcome and a sense of good alignment were the main factors correlating with better HRQOL. Positive patient assessments of surgical outcomes may facilitate better post-operative HRQOL.
